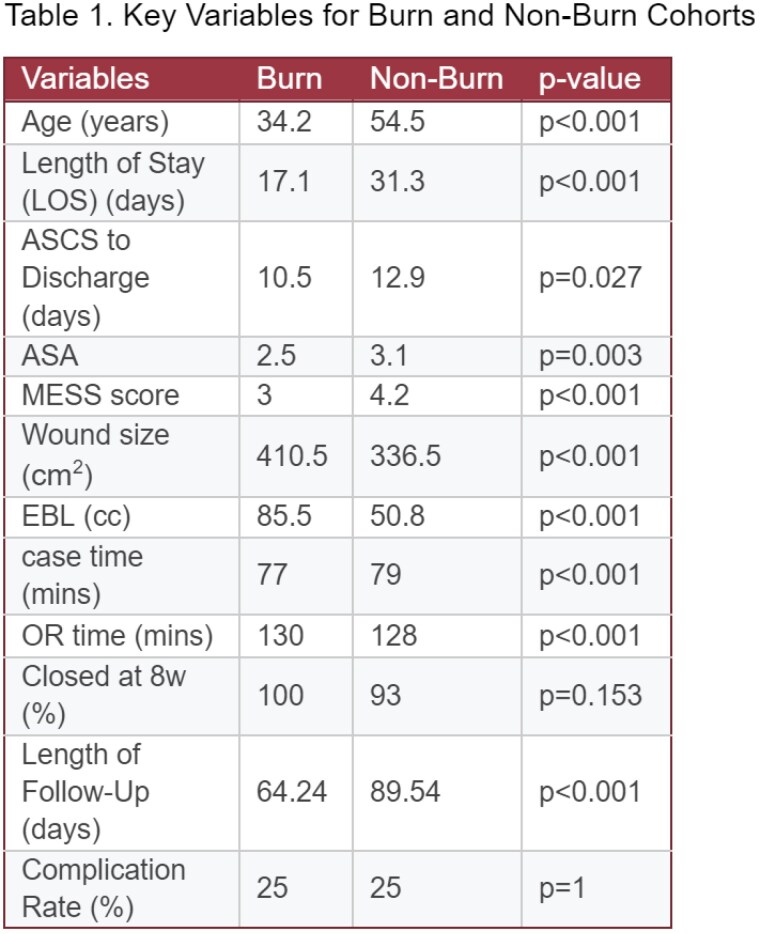# 525 Autologous Skin Cell Suspension Provides Comparable Healing in Burn and Non-Burn Wounds

**DOI:** 10.1093/jbcr/iraf019.154

**Published:** 2025-04-01

**Authors:** Riley Shegos, Sarah Miller, Ursula Adams, Cori Rogers, Carrie Mcgroarty, C Scott Hultman

**Affiliations:** Campbell University School of Osteopathic Medicine; Campbell University School of Osteopathic Medicine; WakeMed Health and Hospitals; WakeMed Health and Hospitals; WakeMed Health and Hospitals; WakeMed Health and Hospitals

## Abstract

**Introduction:**

Burn injuries often require advanced treatments to optimize healing, but the comparative effectiveness of emerging therapies, such as autologous skin cell suspension (ASCS), between wound types remains unclear. While ASCS has shown promise in enhancing wound healing, there is a gap in understanding its relative efficacy in burn versus non-burn wounds. This study evaluates and compares the outcome of ASCS treatment in burn and non-burn patients with the hypothesis that ASCS is equally effective in promoting healing across both wound types.

**Methods:**

This retrospective cohort study analyzed 100 consecutive patients treated with ASCS for full-thickness injuries. We compared burn (n=28) with non-burn (n=72) patients, focusing on age, length of stay (LOS), time from ASCS application to discharge, ASA score, Mangled Extremity Severity Score (MESS), wound size, estimated blood loss (EBL), case time, OR time, wound closure at 4 and 8 weeks, follow-up duration, and complication rates. Statistical significance was assigned for p values < 0.05, using T tests and Chi-square analysis.

**Results:**

At 4 weeks, wound closure was similar for burns (78.6%, 22/28) and non-burns (75%, 54/72). At 8 weeks, closure was 100% (28/28) for burns and 93% (67/72) for non-burns. Complication rates were 25% in both groups (7/28 burn and 18/72 non-burn). Follow-ups averaged 64.24 days (burn) and 89.54 days (non-burn). See Table for additional comparisons.

**Conclusions:**

Comparisons between the ASCS-treated burn and non-burn cohorts showed no statistically significant differences in wound closure or complications, indicating similar efficacy of ASCS for both types of injuries. Overall, these findings suggest that ASCS is a versatile and valuable addition to current burn treatment protocols, offering promising results irrespective of the injury’s etiologies.

**Applicability of Research to Practice:**

The demonstrated efficacy of ASCS can serve as a basis for developing clinical guidelines and protocols allowing clinicians to confidently apply it not only for burns but also for a range of other skin injuries, knowing that it yields favorable results regardless of etiology and allows them to provide the best possible care to their patients.

**Funding for the Study:**

N/A